# Genome-Wide Placental Gene Methylations in Gestational Diabetes Mellitus, Fetal Growth and Metabolic Health Biomarkers in Cord Blood

**DOI:** 10.3389/fendo.2022.875180

**Published:** 2022-05-26

**Authors:** Wen-Juan Wang, Rong Huang, Tao Zheng, Qinwen Du, Meng-Nan Yang, Ya-Jie Xu, Xin Liu, Min-Yi Tao, Hua He, Fang Fang, Fei Li, Jian-Gao Fan, Jun Zhang, Laurent Briollais, Fengxiu Ouyang, Zhong-Cheng Luo

**Affiliations:** ^1^ Ministry of Education-Shanghai Key Laboratory of Children’s Environmental Health, Early Life Health Institute, and Department of Pediatrics, Xinhua Hospital, Shanghai Jiao-Tong University School of Medicine, Shanghai, China; ^2^ Lunenfeld-Tanenbaum Research Institute, Prosserman Centre for Population Health Research, Department of Obstetrics and Gynecology, Mount Sinai Hospital, Faculty of Medicine, University of Toronto, Toronto, ON, Canada; ^3^ Clinical Skills Center, School of Clinical Medicine, Shandong First Medical University & Shandong Academy of Medical Sciences, Jinan, China; ^4^ Department of Obstetrics and Gynecology, Xinhua Hospital, Shanghai Jiao-Tong University School of Medicine, Shanghai, China; ^5^ Department of Obstetrics and Gynecology, Ruijin Hospital, Shanghai Jiao Tong University School of Medicine, Shanghai, China; ^6^ Center for Fatty Liver, Shanghai Key Lab of Pediatric Gastroenterology and Nutrition, Department of Gastroenterology, Xinhua Hospital, Shanghai Jiao Tong University School of Medicine, Shanghai, China; ^7^ Lunenfeld-Tanenbaum Research Institute, Prosserman Centre for Population Health Research, Institute of Health Policy, Management and Evaluation, Dalla Lana School of Public Health, University of Toronto, Toronto, ON, Canada

**Keywords:** GDM, fetal growth, placental DNA methylations, cord blood biomarkers, birth weight

## Abstract

Gestational diabetes mellitus (GDM) “program” an elevated risk of metabolic syndrome in the offspring. Epigenetic alterations are a suspected mechanism. GDM has been associated with placental DNA methylation changes in some epigenome-wide association studies. It remains unclear which genes or pathways are affected, and whether any placental differential gene methylations are correlated to fetal growth or circulating metabolic health biomarkers. In an epigenome-wide association study using the Infinium MethylationEPIC Beadchip, we sought to identify genome-wide placental differentially methylated genes and enriched pathways in GDM, and to assess the correlations with fetal growth and metabolic health biomarkers in cord blood. The study samples were 30 pairs of term placentas in GDM vs. euglycemic pregnancies (controls) matched by infant sex and gestational age at delivery in the Shanghai Birth Cohort. Cord blood metabolic health biomarkers included insulin, C-peptide, proinsulin, IGF-I, IGF-II, leptin and adiponectin. Adjusting for maternal age, pre-pregnancy BMI, parity, mode of delivery and placental cell type heterogeneity, 256 differentially methylated positions (DMPs,130 hypermethylated and 126 hypomethylated) were detected between GDM and control groups accounting for multiple tests with false discovery rate <0.05 and beta-value difference >0.05. WSCD2 was identified as a differentially methylated gene in both site- and region-level analyses. We validated 7 hypermethylated (CYP1A2, GFRA1, HDAC4, LIMS2, NAV3, PAX6, UPK1B) and 10 hypomethylated (DPP10, CPLX1, CSMD2, GPR133, NRXN1, PCSK9, PENK, PRDM16, PTPRN2, TNXB) genes reported in previous epigenome-wide association studies. We did not find any enriched pathway accounting for multiple tests. DMPs in 11 genes (CYP2D7P1, PCDHB15, ERG, SIRPB1, DKK2, RAPGEF5, CACNA2D4, PCSK9, TSNARE1, CADM2, KCNAB2) were correlated with birth weight (z score) accounting for multiple tests. There were no significant correlations between placental gene methylations and cord blood biomarkers. In conclusions, GDM was associated with DNA methylation changes in a number of placental genes, but these placental gene methylations were uncorrelated to the observed metabolic health biomarkers (fetal growth factors, leptin and adiponectin) in cord blood. We validated 17 differentially methylated placental genes in GDM, and identified 11 differentially methylated genes relevant to fetal growth.

## Introduction

Gestational diabetes mellitus (GDM) is a common pregnancy complication (1). The offspring of GDM mothers are at an increased risk of metabolic syndrome (2). The mechanisms remain unclear. Epigenetic alterations are a plausible mechanism in such fetal “programming”.

Placenta is a fetal organ for nutrient and waste exchanges between the mother and the fetus, and plays a pivotal role in fetal growth and development (3). A number of studies have reported placental epigenetic changes in maternal hyperglycemia *via* candidate gene approach measuring proximal promoter region methylation levels in metabolic health-relevant genes (e.g. leptin, adiponectin, lipoprotein lipase) (4–10). However, later epigenome-wide association studies failed to confirm these findings (11–17). Furthermore, there is a lack of consistent findings in epigenome-wide association studies, probably partly due to relatively small sample sizes and differences in study population, data calibration or statistical analysis method, and definition of GDM (16). Some studies have reported altered methylations in some pathways related to metabolic diseases or fetal development (11, 12, 14, 17), suggesting that placental epigenetic dysregulation may reflect fetal programming effects. We are aware of only one study controlling for placental cell heterogeneity in placental epigenome-wide association analyses in GDM (15). The placental methylome represents a combination of signals from different cell types, and cell type composition/variation (18) would affect gene-level methylation data. Little is known about whether any GDM associated differentially methylated genes are relevant to fetal growth or circulating (cord blood) metabolic health biomarkers.

In the present study, we sought to assess GDM-associated genome-wide DNA methylation changes to identify replicable differentially methylated genes and enriched pathways vs. the literature. We also sought to examine the biological significance of differentially methylated genes through assessing the correlations with fetal growth (birth weight) and metabolic health biomarkers in cord blood.

## Methods

### Subjects, Data and Specimens

We randomly sampled 30 pairs of GDM and controls in term births with placenta and cord blood specimens available for assays in the Shanghai Birth Cohort (SBC) (19, 20). Briefly, the SBC recruited 4127 women during preconception and early pregnancy care visits during 2013-2016 in six university-affiliated tertiary obstetric care hospitals in Shanghai. Women were followed up at the first, second and third trimesters of pregnancy and delivery, with data and specimens collected at each study visit. The project was approved by the research ethics committees of Xinhua Hospital, Shanghai (the coordination center, ref no. M2013-010) and all participating hospitals.

All participants received a 75-g oral glucose tolerance test (OGTT) at 24-28 weeks of gestation, and GDM was diagnosed according to the International Association of Diabetes and Pregnancy Study Group (IADPSG) criteria (blood glucose concentration >=anyone of the following thresholds: fasting 5.1 mmol/L, 1-hour 10.0 mmol/L and 2-hour 8.5 mmol/L) (21). The present study included 30 GDM (5 with insulin treatment, 25 with dietary/lifestyle interventions) and 30 euglycemic controls matched by fetal sex and gestational age at delivery (within 1 week). All cases and controls were term births with normal Apgar score (>7), and all mothers were free of severe chronic diseases before pregnancy (e.g., essential hypertension, chronic diabetes) or severe pregnancy complications (e.g. preeclampsia).

Birth weight was standardized to z score according to the 2015 Chinese sex- and gestational age-specific birth weight standards (22). Maternal pre-pregnancy body mass index (BMI) (kg/m^2^) was categorized by Chinese standards (<18.5 underweight; 18.5-23.9 normal weight; 24.0+ overweight/obesity) (23). Other maternal and infant characteristics included maternal ethnicity (all Han Chinese), age (years), education (university: yes/no), parity (primiparity: yes/no), family history of diabetes in first-degree relatives (yes/no), mode of delivery (cesarean section/vaginal), infant sex and gestational age (week) at birth.

Cord blood and placenta specimens were collected immediately after delivery. Cord blood samples were centrifuged within 2 hours of specimen collection at 4°C, 4000 r/min for 10 min. Serum (without any anticoagulant) and EDTA plasma aliquots were stored at -80°C until assays. Each of the four placenta quadrants was sampled approximately 1.5 cm away from the umbilical cord insertion from the fetal side of the placenta. Fetal membrane and visible large vessels were removed, and phosphate-buffered saline (PBS) was used to wash placenta samples before separating into maternal- and fetal-side samples. Samples were stored at -80°C until DNA extraction. DNA was extracted from fetal-side placental samples using DNeasy Blood & Tissue Kit (Qiagen, Cat No. 69504). Purity was examined by A_260_:A_280_ ratio which ranged between 1.87 and 1.90.

### Epigenome-Wide DNA Methylation Measurements

Placental DNA methylations at more than 850,000 CpG sites across the genome were measured by Infinium MethylationEPIC BeadChip (Illumina, San Diego, CA). Samples were randomly placed in different slides (total 12) in the assays. Package “minfi” was used to import and preprocess raw methylation data in R. For all the samples, the proportion of CpG sites with detection P value >0.01 was less than 5%. Thus, all samples were included in subsequent data analyses. We excluded 2677 CpG sites with detection P value >0.01 in more than 5% of all the samples. Functional normalization was applied to remove between-array (unwanted) variations using control probes (24). We excluded 41710 CpG sites with bead count less than 3 in more than 5% of all samples, 18487 annotated to sex chromosomes, 83460 SNPs inside the probe body, in the CpG interrogation, or at the single nucleotide extension with a minor allele frequency of ≥0.05, 33202 suspected cross-reactive sites (25), and 2301 non-CpG sites, leaving 684022 CpG sites in further data analysis. Beta-mixture quantile dilation (BMIQ) was then applied to adjust for type-2 probe bias (26). We excluded sites with average methylation levels <5% or >95% (n=183267), as extreme β values tended to have low reproducibility (27), and small to moderate changes in methylation levels may not have any significant biological implication at both extremes. A total of 500755 CpG sites were retained in the final epigenome-wide association analysis. Potential bias due to different slides was adjusted for by the ComBat function in sva package (28). The density plot did not reveal significant differences in beta value distributions between GDM and control groups (**Figure S1**). Beta values were transformed to M values using lumi package for differential methylation analysis since M values had better statistical performance (29). Beta values were presented for quantifying the differences in methylation levels between groups for ease of data interpretation.

### Bisulfite Sequencing Validation Study

We sought to validate a few selected DMPs with relatively larger methylation differences between GDM and control groups in the epigenome wide association analysis. The study subjects were an independent random sample of 47 pairs of GDM and controls from the Shanghai Birth Cohort. 4 DMPs (CpG sites) were chosen as the corresponding delta betas were in the top 30 DMPs and the corresponding genes have been related to glucose homeostasis (30, 31). These CpG sites were annotated to WSC Domain Containing 2 (WSCD2), phosphodiesterase 1C (PDE1C), and protocadherin Beta 15 (PCDHB15). DNA was sodium-bisulfite treated (EZ DNA Methylation-Lighting) and PCR-amplified with primers designed by PyroMark Assay Design software (version 2.0; Qiagen). Pyrosequencing was performed using PyroMark Q48 (Qiagen, appendix –Methodology in the pyrosequencing study).

### Biochemical Assays

Cord serum insulin and insulin-like growth factor 1 (IGF-I) concentrations were measured by chemiluminescent assays (ADVIA Centaur and Immulite2000, SIEMENS, Germany). Cord plasma C-peptide and proinsulin were measured by enzyme-linked immunosorbent assay (ELISA) kits from Mercodia (Uppsala, SE). IGF-II was measured by an ELISA kit from R&D system (Minnesota, USA). Plasma high-molecular-weight (HMW) and total adiponectin concentrations were measured by an ELISA kit from ALPCO (Salem, NH, USA), and plasma leptin by an ELISA kit from Invitrogen (Carlsbad, CA, USA), respectively. The detection limits were 3.5 pmol/l for insulin, 25 pmol/L for C-peptide, 1.7 pmol/l for proinsulin, 25 ng/ml for IGF-I, 1.88 pg/ml for IGF-II, 0.034 ng/ml for HMW and total adiponectin, and 3.5 pg/ml for leptin, respectively. Intra-assay and inter-assay coefficients of variation were in the ranges 0.4-13.5% (32, 33). The laboratory technicians were blinded to the clinical status of study participants.

### Statistical Analysis

All analyses were conducted in R Studio (version: 2021.09.1). The associations between GDM status and gene DNA methylation levels (M-values) were assessed by lmFit function in R limma package. For placental cell types, we used ReFactor to select the number of principal components accounting for a substantial proportion of the variance in the data, and then compute the selected 4 components in adjusting for cell-type heterogeneity in differential methylation position (DMP) analyses (34). The comparisons were adjusted for important covariates including maternal age, pre-pregnancy body mass index, primiparity (yes/no), infant sex, gestational age at birth and mode of delivery (vaginal/caesarean section). To minimize false discovery findings, p values were adjusted according to Benjamini and Hochberg’s method accounting for multiple tests. DMPs were selected at false discovery rate (FDR) threshold of 0.05 and absolute methylation difference (delta beta) >0.05 (to identify “true” DMPs with differences that are unlikely be measurement errors) (16). To explore the functional roles of the DMPs, we performed Ingenuity Pathway Analysis to annotate the most significant canonical pathways.

Differentially methylated regions (DMRs) were identified using comb-p (35) and DMRcate package (36). Comb-p deals with autocorrelations in neighboring P values and reports region-based P values with Sidak correction for multiple tests. P value <10^-3^ was set to start a region and a distance of 200 bp was selected to extend the region in the presence of another P value <10^-3^. DMRcate package identified the most differentially methylated regions based on tunable kernel smoothing of the signal of methylation changes (36). As recommended by the authors, a bandwidth of 1000 nucleotides and a scaling factor of 2 were used. Significant DMRs were selected at FDR <0.05.

Partial correlation analyses were conducted to examine the associations with cord blood metabolic health biomarkers (insulin, proinsulin, C-peptide, IGF-I, IGF-II, leptin, adiponectin) adjusting for gestational age at birth. First, we assessed the correlations with DNA methylation levels in the respective specific genes (LEP, ADIPOQ, IGF1, INS-IGF2). Of the 500755 CpG sites, 21 sites were annotated to LEP (chr7: 127876829-127894849), 12 sites to ADIPOQ (chr3: 186559147-186575325), 12 sites to IGF1 (chr12: 102795843-102875301) and 119 sites to INS/INS-IGF2/IGF2 (chr11: 2150687- 2183864). For each gene, mean methylation levels were computed for moderately/highly correlated sites (r>0.5). Benjamini-Hochberg method was used in calculating the adjusted P values accounting for multiple tests. Second, we assessed the correlations of GDM-associated DMPs with birth weight (z score) and cord blood metabolic health biomarkers (in log-transformed data). CpG sites with crude P values <0.05 were presented along with the Benjamini-Hochberg adjusted P values accounting for multiple tests.

## Results

### Characteristics of Study Subjects

Comparing GDM vs. controls (n=30 pairs), women with GDM had higher pre-pregnancy BMI (24.4 ± 3.5 vs. 21.2 ± 3.8 kg/m^2^), and were more likely to be overweight or obese (57.7% vs 18.5%), or to have a caesarean section delivery (73.3% vs. 26.7%) **(** **Table 1** **)**. As expected, women with GDM had higher fasting, 1h and 2h blood glucose levels in the 75-OGTT tests. All women were non-smokers. There were no significant differences in maternal age, education, parity, family history of diabetes, alcohol drinking during pregnancy and gestational weight gain. Comparing the infants of women with GDM vs. euglycemic pregnancies, birth weight z scores were substantially higher (1.23 ± 1.39 vs. 0.13 ± 0.66).

**Table 1:** Maternal and neonatal characteristics in a matched study of 30 pairs of term placentas in gestational diabetes mellitus (GDM) vs. euglycemic (control) pregnancies in Shanghai birth cohort.

	GDM (n=30)	Control (n=30)	P*
**Mothers**			
Age	29.8 ± 4.1	29.8 ± 3.2	0.999
>35 years	4 (13.3)	2 (6.7)	0.335
Alcohol drinking in pregnancy	2 (7.7)	7 (25.9)	0.079
Family history of diabetes	6 (20.0)	2 (6.7)	0.127
Education, university	18 (60.0)	20 (66.7)	0.592
Primiparity	26 (86.7)	24 (80.0)	0.757
Prepregnancy BMI (kg/m^2^)	24.4 ± 3.48	21.2 ± 3.78	**0.002**
BMI group			**0.023**
<18.5	1 (3.8)	5 (18.5)	
18.5-24.0	10 (38.5)	17 (63.0)	
24.0+	15 (57.7)	5 (18.5)	
Gestational weight gain (kg)	14.9 ± 4.8	15.9 ± 4.9	0.475
Z score	0.17 ± 0.81	0.19 ± 0.99	0.920
Caesarean section	22 (73.3)	8 (26.7)	**<0.001**
75 g OGTT glucose (mmol/L)			
Fasting	4.9 ± 0.6	4.4 ± 0.4	**<0.001**
1-hour	9.8 ± 1.7	7.0 ± 1.5	**<0.001**
2-hour	7.9 ± 2.0	6.0 ± 1.2	**<0.001**
HbA1c (%) at 32-35 weeks	5.3 ± 0.4	5.1 ± 0.3	0.061
**Newborns**			
Birth weight (g)	3814 ± 524	3390 ± 264	**<0.001**
Z score	1.23 ± 1.39	0.13 ± 0.66	**<0.001**
Birth length (cm)	50.5 ± 1.14	50.05 ± 0.68	0.076
Z score	0.51 ± 1.13	0.03 ± 0.71	0.060
Sex, boy	17 (56.7)	17 (56.7)	1.000
Gestational age at delivery	39.4 ± 0.68	39.6 ± 0.80	0.185
Cord blood biomarkers			
Insulin (pmol/L)	32.7 ± 28.9	40.1 ± 43.4	0.716
C-peptide (pmol/L)	284.5 ± 170.6	286.8 ± 161.1	0.994
Proinsulin (pmol/L)	26.9 ± 22.4	24.4 ± 22.1	0.920
Leptin (ng/ml)	9.3 ± 7.7	9.4 ± 6.7	0.407
Adiponectin-HMW (µg/ml)	14.9 ± 8.9	20.5 ± 10.4	**0.018**
Adiponectin-Total (µg/ml)	32.8 ± 14.9	40.6 ± 18.4	0.073
IGF-I (ng/ml)	81.6 ± 27.9	68.7 ± 22.9	0.083
IGF-II (ng/ml)	198.5 ± 28.3	191.4 ± 32.8	0.335

Data presented are mean ± SD or n (%); HMW, high-molecular-weight.

^*^P values for differences between the two groups; P values in bold, P<0.05.

Cord plasma high molecular weight adiponectin concentrations were lower in GDM vs. controls (14.9 ± 8.9 vs. 20.5 ± 10.4 µg/ml, P=0.015), while the differences in cord blood insulin, C-peptide, proinsulin, IGF-I, IGF-II and leptin concentrations were not statistically significant.

### Differentially Methylated Positions

Adjusting for maternal age, pre-pregnancy BMI, parity, infant sex, gestational age, mode of delivery and the four principal components representing placental cell type proportions, at FDR <0.05 and delta beta >0.05, we identified 256 DMPs (130 hypermethylated and 126 hypomethylated) between GDM and control groups. These loci were distributed over 133 genes (71 genes in hypermethylated loci, 64 genes in hypomethylated loci; 3 genes were in both) (**Table S3**). Epigenome-wide association results were shown in **Figure S2**. The mean ± SD of the methylation differences between GDM and control groups were 7.5 ± 2.7% and 7.2 ± 2.5% for hypermethylated and hypomethylated loci, respectively.

The top 30 DMPs (15 hypermethylated, 15 hypomethylated) are presented in **Table 2**. The β value differences (Δβ) in the 15 hypermethylated sites range from 0.11 to 0.18. These loci were annotated to 12 genes (PCDHB15, DKK2, FAM65B, CYP26C1, MIATNB, ERG, CADM2, GFRA1, DPYD, HCN1, C14orf39, and SLC9A9). The Δβ of the top 15 hypomethylated sites range from -0.18 to -0.10, and these sites were annotated to 8 genes (DPP10, PENK, TSNARE1, WSCD2, LOC102723828, NRXN1, PDE1C and MIR124-2).

**Table 2:** Top 30 differentially methylated positions (15 hypermethylated and 15 hypomethylated DMPs) in placental DNAs in GDM vs. euglycemic (control) groups.

CpG site	Chr	Gene	Gene group	Beta (GDM)	Beta (Control)	Delta beta	FDR
**Hypermethylated**							
cg06295987	chr3			0.323	0.14	0.183	0.007
cg26380443	chr5	PCDHB15	1stExon	0.388	0.212	0.176	0.046
cg17010273	chr4	DKK2	TSS1500	0.373	0.205	0.169	0.019
cg03975200	chr6	FAM65B	TSS1500	0.711	0.546	0.165	0.047
cg07204602	chr10	CYP26C1	TSS1500	0.752	0.601	0.152	0.048
cg11423008	chr22	MIATNB	Body	0.775	0.627	0.149	0.024
cg15480311	chr21	ERG	Body; 5’UTR	0.519	0.391	0.128	0.039
cg25429559	chr3	CADM2	5’UTR;Body	0.858	0.732	0.126	0.041
cg06039355	chr10	GFRA1	5’UTR;TSS1500	0.441	0.321	0.12	0.007
cg26836456	chr1	DPYD	TSS1500	0.507	0.388	0.119	0.049
cg18414238	chr5	HCN1	Body	0.681	0.569	0.112	0.048
cg12189551	chr14	C14orf39	TSS200	0.261	0.15	0.112	0.042
cg16079012	chr3	SLC9A9	Body	0.744	0.635	0.109	0.049
cg17943876	chr5			0.552	0.445	0.107	0.007
cg05684050	chr5			0.775	0.67	0.106	0.04
**Hypomethylated**							
cg16863522	chr2	DPP10	3’UTR	0.274	0.454	-0.18	0.017
cg27531336	chr8	PENK	TSS1500	0.276	0.433	-0.157	0.037
cg13112267	chr8	TSNARE1	Body	0.642	0.799	-0.157	0.046
cg13713677	chr12	WSCD2	TSS200	0.182	0.334	-0.152	0.028
cg05041928	chr4	LOC102723828	Body	0.561	0.697	-0.136	0.036
cg08664343	chr21			0.563	0.693	-0.13	0.028
cg26852437	chr11			0.561	0.69	-0.128	0.012
cg05581275	chr2	DPP10	Body	0.291	0.406	-0.115	0.012
cg13594075	chr2	NRXN	TSS200	0.411	0.526	-0.114	0.026
cg01097881	chr12	WSCD2	TSS200	0.25	0.362	-0.112	0.012
cg19502018	chr7	PDE1C	Body	0.583	0.695	-0.111	0.011
cg04831599	chr8			0.377	0.486	-0.109	0.03
cg24118713	chr12			0.634	0.741	-0.106	0.01
cg05474726	chr8	MIR124-2	TSS200	0.269	0.375	-0.106	0.04
cg10104921	chr4			0.491	0.596	-0.104	0.028

FDR, false discovery rate accounting for multiple tests.

### Pyrosequencing Validation Study in an Independent Sample

The differences in methylation levels between GDM and control groups in the pyrosequencing validation study (n=47 GDM-control pairs) and Infinium MethylationEPIC BeadChip (850K) study are in the same direction for all the four DMPs in the three genes (WSCD2, PDE1C, and PCDHB15) (**Table S1**), although the methylation differences between the two groups did not reach statistical significance in the pyrosequencing study.

### Validated Differentially Methylated Genes vs. the Literature

The validated placental differentially methylated genes vs. the literature are listed in **Table S2**. Our MethylationEPIC BeadChip data validated 7 hypermethylated genes (CYP2D7P1, GFRA1, HDAC4, LIMS2, NAV3, PAX6 and UPK1B), and 10 hypomethylated genes (DPP10, CPLX1, CSMD2, GPR133, NRXN1, PCSK9, PENK, PRDM16, PTPRN2 and TNXB) reported in previous placenta epigenome-wide association studies in GDM (11, 13, 16).

### Ingenuity Pathway Analysis

IPA analysis of DMPs identified 10 canonical pathways with Fisher’s exact test crude P<0.05 (**Table 3**). However, none of the pathways achieved statistical significance after correction for multiple tests. Differentially methylated genes were most enriched in bladder cancer signalling (4 genes, crude P=0.005), followed by GABA receptor signalling (4 genes, crude P=0.008) and uracil degradation II (reductive) (1 gene, crude P=0.023).

**Table 3:** Canonical pathways of differentially methylated positions (DMPs) in placental genes in GDM vs. control groups.

Pathway	P*	Overlapped genes (ratio)
Bladder Cancer Signaling	0.005	4/114 (0.035)
GABA Receptor Signaling	0.008	4/130 (0.0308)
Uracil Degradation II (Reductive)	0.023	1/4 (0.25)
Thymine Degradation	0.023	1/4 (0.25)
CREB Signaling in Neurons	0.025	8/591 (0.0135)
tRNA Splicing	0.028	2/44 (0.0455)
Gustation Pathway	0.030	4/197 (0.0203)
Amyotrophic Lateral Sclerosis Signaling	0.031	3/114 (0.0263)
Cardiac Hypertrophy Signaling (Enhanced)	0.039	7/527 (0.0133)
cAMP-mediated signaling	0.047	4/226 (0.0177)

^*^Crude p value from Fisher’s exact test.

### Differentially Methylated Regions

In search of genomic regions where methylation levels differ between GDM and control groups, comb-p identified 4 DMRs (**Table S4**) with Sidak corrected P<0.05. Two DMRs were annotated to PCSK9 and WSCD2D, while the other two DMRs were located in the OpenSea area. DMRcate identified 42 DMRs at FDR <0.05 that were annotated to 29 genes spanning 2 to 13 CpG sites (**Table S5**). The most significant region was located in the promoter and the first exon of WSCD2 in chromosome 12, containing 13 CpG sites. Two overlapped DMRs were identified by both DMRcate and comb-p. One DMR (Chr12:108522704-108524345) was located in the WSCD2 promoter and the other (Chr5: 92275312-92275387) in the OpenSea area (**Table 4**). The methylation levels in the GDM group were lower in the WSCD2 promoter region.

**Table 4:** Differentially methylated regions (DMRs, GDM vs. control groups) identified by both comb-p and DMRcate package in region-level data analyses in R.

DMR	CpGs comprising the DMR	Direction of association	Gene	Gene group/Relation to island
**comb-p**				
Chr 5:92275335-92275388	cg21937916, cg19564029	+		OpenSea
Chr 12:108523395-108523473	cg01097881, cg13236378, cg05062612, cg26373942, cg06326926	–	WSCD2	TSS200/Island
**DMRcate**				
Chr 5: 92275312-92275387	cg14411504, cg19564029, cg21937916	+		OpenSea
chr12:108522704-108524345	cg12728312, cg22330512, cg13713677, cg08047802, cg03111482, cg01097881, cg13236378, cg05062612, cg26373942, cg06326926, cg15873673, cg10419678 cg03626024	–	WSCD2	TSS1500, TSS200, 5’UTR,1stExon/N_Shore, Island, S_Shore

GDM, gestational diabetes mellitus.

### Gene-Specific Correlations Between Placental DNA Methylations and Cord Blood Biomarkers

**Table S6** presents gene-specific correlations of placental gene DNA methylations with cord blood biomarkers. The total numbers of sites in the Pearson partial correlation analyses were 17 for LEP, 12 for ADIPOQ, 7 for IGF1 and 71 for INS/INS-IGF2/IGF2 genes, respectively. For LEP, methylation at cg05136031 was positively correlated with cord blood leptin concentration (r=0.28, crude P=0.033). For INS-IGF2/IGF2/INS, methylation levels in two CpG sites (cg17434309, cg10650127) were positively correlated with cord blood insulin (r=0.32 and P=0.009, r=0.29 and P=0.028, respectively). Methylation at cg17434309 was correlated with C-peptide. Methylation levels in one site and one region (cg13670288, mean of cg27331871 and cg25742037) were negatively correlated with cord blood proinsulin. Methylation levels in three CpG sites and one region (cg23889607, cg24366657, cg25163476, mean of cg02749887 and cg21574853) were positively correlated with IGF2. However, all these correlations did not reach statistical significance after correction for multiple comparisons.

### Correlations of Placental DMPs With Birth Weight and Cord Blood Biomarkers

Among the 256 DMPs, 38 DMPs (34 genes) were correlated with birth weight (z score) at crude P<0.05, and the correlations remained statistically significant for 12 DMPs (11 genes) after BH correction for multiple tests (**Table 5**). Methylations in seven genes (PCDHB15, DKK2, ERG, CADM2, CYP2D7P1, SIRPB1, KCNAB2) were positively correlated with birth weight with BH adjusted P <0.05. Methylations in four genes (RAPGEF5, CACNA2D4, PCSK9, TSNARE1) were negatively correlated with birth weight with BH adjusted P<0.05.

**Table 5:** Correlations of birth weight (z score) with differentially methylated genes.

Gene	probeID	r	P	BH corrected P*
CYP2D7P1	cg26169700	0.432	0.00056	0.04096
PCDHB15	cg26380443	0.420	0.00084	0.04096
ERG	cg15480311	0.416	0.00096	0.04096
SIRPB1	cg11330406	0.414	0.001	0.04096
DKK2	cg17010273	0.401	0.00152	0.04096
RAPGEF5	cg24092655	-0.399	0.00157	0.04096
RAPGEF5	cg20583679	-0.389	0.00213	0.04651
CACNA2D4	cg10719390	-0.388	0.00218	0.04651
PCSK9	cg17167852	-0.382	0.00258	0.04722
TSNARE1	cg13112267	-0.378	0.00289	0.04722
CADM2	cg25429559	0.375	0.00317	0.04722
KCNAB2	cg00813560	0.371	0.00352	0.04743
DPYSL2	cg06601131	0.362	0.00449	0.05225
NME7	cg16468828	0.348	0.00647	0.06370
LOC101927292	cg05401617	-0.341	0.00759	0.06939
LAIR2	cg20313638	0.34	0.00795	0.07018
PDE1C	cg19502018	-0.334	0.00919	0.07842
CCT4	cg12168100	0.331	0.0098	0.08093
CD200R1L	cg24025902	-0.328	0.01046	0.08114
UPK1B	cg26433505	0.317	0.01364	0.09563
KIAA1429	cg17966245	0.315	0.01419	0.09563
MIATNB	cg11423008	0.312	0.01512	0.09563
C3P1	cg12272488	0.312	0.01537	0.09563
MBP	cg12979350	-0.311	0.01569	0.09563
NAV3	cg20297402	0.307	0.01708	0.10169
PLIN5	cg02593507	-0.299	0.02038	0.11253
PCSK9	cg09072162	-0.294	0.02244	0.11489
MIATNB	cg07281538	0.289	0.02504	0.12569
C4orf37	cg08746785	0.285	0.02716	0.13119
OBSCN	cg02281446	-0.280	0.03016	0.13811
C1orf114	cg06783102	-0.279	0.03071	0.13811
NACC2	cg24597547	-0.277	0.03197	0.14111
PHACTR3	cg08481633	0.269	0.03744	0.15819
GABRA5	cg00796569	0.264	0.04151	0.16604
CFAP70	cg24284700	0.261	0.04364	0.17187
PTPRN2	cg24764310	-0.260	0.04461	0.17303
NACC2	cg15344596	-0.259	0.04533	0.17320
FRMD6	cg09376996	0.256	0.04875	0.18353

^*^P value with Benjamini-Hochberg correction for multiple tests.

In regression analyses, the positive association between GDM and birth weight (β=1.10, P<0.01) became non-significant after adjusting for the methylation levels in these 38 DMPs (β=-0.76, P=0.42). These 11 genes were enriched in nNOS signaling in skeletal muscle cells (Fisher’s exact crude P=0.024), netrin signaling (P=0.037), maturity onset diabetes of young signaling (P=0.039), and FcγRIIB signaling in B lymphocytes (P=0.042).

Placental gene DMPs that were significantly correlated with one or more cord blood biomarkers (leptin, total and HMW-adiponectin, insulin, proinsulin, C-peptide, IGF1 and IGF2) at crude P <0.05 are presented in **Table S7**. Notably, PAX6 (cg09382096) methylation was negatively correlated with cord blood leptin (r=-0.34, P=0.01), total adiponectin (r=-0.30, P=0.02) and HMW-adiponectin (r=-0.39, P=0.003). SLC9A3 (cg02689506) methylation was negatively correlated with cord blood leptin (r=-0.31, P=0.02), total adiponectin (r=-0.32, P=0.014) and HMW-adiponectin (r=-0.44, P=0.001). WSCD2 (cg15873673) methylation was positively correlated with cord blood total adiponectin (r=0.30, P=0.02), HMW-adiponectin (r=0.28, P=0.036), but negatively correlated with insulin (r: -0.29 to -0.32, P<0.05) and C-peptide (r: -0.29 to -0.31, P<0.05). FGF19 (cg18210732) methylation was negatively correlated with cord blood leptin (r=-0.30, P=0.02) and HMW-adiponectin (r=-0.28, P=0.034). However, none of these correlations reached statistical significance after correction for multiple tests.

## Discussion

### Main Findings

This study identified 256 GDM-associated placental DNA DMPs, and validated 17 differentially methylated genes reported in previous epigenome-wide association studies. The WSCD2 gene was identified as differentially methylated in both site- and region-level analyses. We discovered methylations in 11 placental genes relevant to fetal growth, but none placental gene methylations were correlated to the observed metabolic health biomarkers (fetal growth factors, leptin and adiponectin) in cord blood accounting for multiple tests.

### Differentially Methylated Positions

We detected 256 DMPs localized on 133 genes, and validated 17 differentially methylated genes (CYP1A2, GFRA1, HDAC4, LIMS2, NAV3, PAX6, UPK1B, DPP10, CPLX1, CSMD2, GPR133, NRXN1, PCSK9, PENK, PRDM16, PTPRN2, TNXB) reported in the literature (11, 13, 16). Several genes have been implicated in glucose metabolism and merit comments. GFRA1 has been related to β-cell proliferation and islet repairing (37). GFRA1 was hypermethylated in GDM placentas in a previous study (13). We observed that a locus of PAX6 was hypermethylated in GDM placentas, consistent with a previous study (16). PAX6 may be involved in the regulation of glucose-insulin homeostasis (38, 39). PCSK9 and PTPRN2 have been associated with lipoprotein metabolism and insulin release, respectively (40–42), and were hypomethylated in our and previous epigenome-wide studies (13, 16).

Our and other studies demonstrated substantial placental epigenetic changes in GDM, but only 17 replicable differentially methylated genes vs. the literature. This may be partly attributable to differences in sample size, GDM definition, and cell heterogeneity in placental sample tissues. DNA methylation changes by high-throughput methods in bulk placental tissues should be interpreted with caution. Validation data from different study cohorts may provide important clues on the true altered gene methylations. Ethnicity may be a factor influencing the reproducibility of epigenetic data. All our study subjects were Chinese. It is noteworthy that our data have validated 17 differentially methylated genes in previous reports in Caucasians (11, 13, 16).

The placenta secretes numerous hormones and factors into maternal circulation affecting maternal metabolic health. It is therefore possible that these GDM associated placental differentially methylated genes may be involved in the development of GDM and related complications. Further studies are warranted to understand the potential roles of these genes in the pathophysiology of GDM, and whether the relevant proteins could be promising molecular targets in the prevention of GDM and related complications.

### Bisulfite-Pyrosequencing Validation Study

Bisulfite-pyrosequencing method is the golden standard to measure DNA methylation. We used an independent sample (n=47 GDM/control pairs) in the pyrosequencing validation study of four DMPs annotated to three genes (WSCD2, PCDHB15, and PDE1C). Although the methylation level differences between GDM and controls in the pyrosequencing validation study were all in the same pattern as the 850K BeadChip discovery study, all the differences were not statistically significant in the pyrosequencing validation study. Possible reasons may be partly related to the complex nature of placental tissues; methylation data were likely influenced by cell type composition which was difficult to adequately account for.

### Differentially Methylated Regions

Two DMRs were identified in both comb-p (35) and DMRcate (36) analyses in our study. One DMR was located in the promoter of WSC Domain Containing 2 (WSCD2). Placenta differentially methylated DNA regions in GDM were not assessed in previous studies. A total of 8 CpGs in the WSCD2 gene were identified as hypomethylated DMPs – a consistent finding between region- and site-based data analysesWSCD2 functions as an integral component of the membrane. A single nucleotide polymorphism (rs59849892) in the intronic region of WSCD2 has been linked to insulin sensitivity (43). A recent study reported that WSCD2 expression in human islet was positively correlated with insulin secretion and negatively correlated with HbA1c, suggesting possible hypermethylation in the WSCD2 gene in islet cells in diabetes status and a role of WSCD2 in glucose metabolism (30). However, the placental WSCD2 gene was observed to be hypomethylated in GDM, underscoring the importance of tissue specificity and heterogeneity considerations in methylation data interpretation. The other DMR was not annotated to any gene, and its implications remain to be understood. In contrast, Howe et al. identified two different DMRs in cord blood DNA methylations in GDM using both comb-p and DMRcate in a pooled data analysis of 3677 mother-infant pairs from 7 pregnancy cohorts (44). One DMR was located in the promoter of OR2L13 which has been associated with autism spectrum disorders, and the other was located in the gene body of CYP2E1 which has been implicated in type 1 and type 2 diabetes (44). The discrepant findings could be due to the different tissues in measuring DNA methylations (cord blood vs placenta in our study).

### Canonical Pathways

Our study identified 9 canonical pathways in the IPA analysis with crude P<0.05 in the Fisher’s exact test. These pathways are not overlapping with those reported pathways in previous studies, such as the Wnt signaling (12, 17), MAPK signaling (12, 14, 17), or insulin signaling pathway (14). However, we did not find all statistically significant pathway after correction for multiple tests. The lack of significance could result from a relatively small number of differentially methylated genes (n=133) for identifying the enriched pathways. It should be noted that previous studies used Fisher’s exact test only in the IPA analysis, without correction for multiple tests. The lack of replicable findings concerning the pathways in our study vs. the literature underscores the need for more and larger studies, and the need for standardized analysis approaches.

### Correlations Between DMPs and Birth Weight or Cord Blood Biomarkers

We observed that methylation levels in 11 placental genes were significantly correlated to birth weight (z score) after correction for multiple tests. Birth weight may partly reflect the impact of GDM on the fetus (45). Our results suggest placental epigenetic alterations may play a mediating role in the impact of GDM on fetal growth. We are aware of only one study on placental DMPs in GDM and birth weight, and the study reported 326 GDM associated genes (11). TSNARE1 was the only common gene to these identified in our study. Surprisingly, DNA methylation levels in the TSNARE1 were positively correlated with birth weight in their study (r=0.47, crude P=0.007) (11), but negatively correlated with birth weight in our study. The inconsistent findings suggest the need for more validation studies. We noted that the previous study did not account for multiple tests in examining these correlations (11).

Of the 11 genes with methylations correlated with fetal growth in our study, methylation levels at the PCSK9 promoter were inversely correlated with birth weight (z score). Consistent with our data, a recent study reported lower cord blood PCSK9 concentrations (could be due to higher methylation levels) in intrauterine growth restricted vs. control newborns (46). PCSK9 may play a role in lipoprotein metabolism (40). Circulating PCSK9 concentrations have been positively correlated with total cholesterol, low-density lipoprotein (LDL) cholesterol, and body mass index (47, 48). DNA methylation levels at cell adhesion molecule 2 (CADM2) were positively correlated with birth weight. CADM2 is predominantly expressed in brain, and regulates energy homeostasis *via* central nervous system (49). CADM2 depletion has been associated with reduced adiposity and improved insulin sensitivity in genetically obese mice models (49). Human and animal studies suggest that CADM2 variations regulate body weight (50, 51). We are unaware of any reports on the other 9 genes associated with fetal growth in our data.

We observed no significant associations between GDM-associated DMPs and cord blood biomarkers after correction for multiple tests. We are unaware of any studies on these correlations. Crude correlation coefficients showed that DNA methylation levels at some loci of WSCD2 were correlated with insulin, C-peptide and HMW-adiponectin concentrations. WSCD2 has been implicated in insulin secretion and insulin sensitivity (30, 43). The crude correlation coefficients showed that DNA methylation levels of PAX6 were correlated with leptin and adiponectin concentrations. PAX6 may regulate insulin transcription (52). However, we did not find any correlation between placental PAX6 and cord blood insulin, C-peptide or proinsulin. FGF19 has been implicated in lipid and glucose metabolism (53). Placental DNA methylation levels in FGF19 were correlated with cord blood leptin and HMW-adiponectin. However, none of these correlations were significant after correction for multiple tests, suggesting the need for more validation studies.

### Correlations Between Gene-Specific Placental DNA Methylation and Cord Blood Biomarkers

Adjusting for multiple tests, we did not find any significant correlation between gene-specific placental DNA methylations and the respective cord blood metabolic health biomarkers including insulin, IGF-1, IGF-2, leptin and adiponectin. We observed a trend towards a positive correlation between methylation in a CpG site in the LEP promoter (cg05136031) and cord blood leptin, consistent with a recent study report (54). The cg05136031 methylation has been negatively correlated with BMI (z score) at age 3 years (54). We did not find any correlation between cg15758240 and cord blood leptin, while the previous study reported a negative correlation that might mediate the association between maternal hyperglycemia and cord blood leptinemia (n=262) (54). The non-significant association in our study may be partly attributable to the relatively small sample size (n=60).

### Strengths and Limitations

The major strengths include the use of the most recent DNA methylation microarray covering 850K+ CpG sites, and the more robust statistical analysis approach adjusting for placental cell heterogeneity. All study subjects were Chinese, minimizing the potential confounding effects due to ethnic variations in DMPs. The study has limitations. First, the study had a moderate sample size, and we could not rule out the possibility of false positive findings. Large studies are warranted to validate the findings. Sub-group analyses by GDM treatment (insulin/diet) were not possible due to small sample size (only 5 GDM patients with insulin treatment). Second, DNA methylation levels from pyrosequencing validation study did not achieve statistically significant differences between GDM and control groups, underscoring the uncertain nature of discoveries from genome-wide association analyses. Third, maternal lifestyle factors including smoking, physical activity and dietary factors may affect placental gene methylations. In our study, all pregnant women were non-smokers. We did not have data on maternal physical activity and dietary factors. Further studies are warranted to understand whether these factors may confound the associations between GDM and placental gene methylations.

In summary, GDM was associated with altered DNA methylations in a number of placental genes, but these placental gene methylations were uncorrelated to the observed metabolic health biomarkers in cord blood (fetal growth factors, leptin and adiponectin). We validated 17 GDM associated differentially methylated placental genes reported in the literature, and discovered 11 differentially methylated genes relevant to fetal growth.

## Data Availability Statement

The datasets presented in this article are not readily available because access to the deidentified participant research data must be approved by the research ethics board on a case-by-case basis. The EWAS data on placental gene methylations are available at https://www.ncbi.nlm.nih.gov/geo/query/acc.cgi?acc=GSE200659. Requests to access the datasets should be directed to Zhong-Cheng Luo (zcluo@lunenfeld.ca) or Fangxiu Ouyang (ouyangfengxiu@xinhuamed.com.cn) for assistance in data access request.

## Ethics Statement

The studies involving human participants were reviewed and approved by the research ethics committees of Xinhua Hospital and all participating hospitals. The patients/participants provided their written informed consent to participate in this study.

## Author Contributions

Z-CL, J-GF, FL, LB, JZ and FO conceived the study. W-JW, M-NY, RH, TZ, QD, Y-JX, XL, M-YT, HH, FF, J-GF, FL, JZ, FO and Z-CL contributed to the acquisition of research data. WJW and RH conducted the literature review, data analysis and drafted the manuscript. All authors contributed in revising the article critically for important intellectual content, and approved the final version for publication.

## Funding

Supported by research grants from the Ministry of Science and Technology of China (2019YFA0802501, 2017YFE0124700), the Shanghai Municipal Health Commission (2020CXJQ01, 2019Y0157), the Shanghai Municipal Science and Technology Commission (19410713500, 21410713500), the National Natural Science Foundation of China (81961128023, 81903323, 81761128035, 81930095 and 82125032), the National Human Genetic Resources Sharing Service Platform (2005DKA21300) and the Canadian Institutes of Health Research (158616, 155955). The funders have no role in all aspects of the study, including study design, data collection and analysis, the preparation of the manuscript and the decision for publication.

## Conflict of Interest

The authors declare that the research was conducted in the absence of any commercial or financial relationships that could be construed as a potential conflict of interest.

## Publisher’s Note

All claims expressed in this article are solely those of the authors and do not necessarily represent those of their affiliated organizations, or those of the publisher, the editors and the reviewers. Any product that may be evaluated in this article, or claim that may be made by its manufacturer, is not guaranteed or endorsed by the publisher.

## Abbreviations

GDM, gestational diabetes mellitus; BMI: Body mass index; OGTT, oral glucose tolerance test; SBC, Shanghai Birth Cohort; IADPSG, International Association of Diabetes and Pregnancy Study Groups; IGF-I, insulin-like growth factor I; IGF-II, insulin-like growth factor II; IPA, Ingenuity pathway analysis; CI, confidence interval; SD, standard deviation.
