# Supplementary material for: Genome-Wide Placental Gene Methylations in Gestational Diabetes Mellitus, Fetal Growth and Metabolic Health Biomarkers in Cord Blood
Source: Front Endocrinol (Lausanne). 2022 May 26;13:875180. doi: 10.3389/fendo.2022.875180 (PMC9204344; doi:10.3389/fendo.2022.875180)
Supplement: Supplementary file 1 [file DataSheet_1.zip › Appendix - Methodology in the pyrosequencing study.DOCX]

**Appendix - Methodology in the pyrosequencing study**

Specific PCR sequencing primers were designed using PyroMark Assay Design 2.0 software (QIAGEN) to amplify the bisulfite modified target regions. Four markers (CpG sites) in 3 genes were to be validated. Each marker was amplified using a set of PCR primers (in the following Table), one of which was biotin-labeled to generate the biotinylated PCR amplicons needed for the downstream pyrosequencing reaction. The target regions were amplified in a singleplex format by utilizing the PyroMark PCR kit (QIAGEN). The PCR products were pyrosequenced using a PyroMark Q48 pyrosequencer (QIAGEN) per the manufacturer’s instructions. PyroMark Q48 software (QIAGEN) was used to calculate the percent methylation at each CpG site.

**Table - Primers in the pyrosequencing validation study**

| Gene | CpG site | Primer | Primer sequence |
| --- | --- | --- | --- |
| WSCD2 | cg01097881 | cg01097881-F | AGGTTTGGTTTAAGGGGTATT |
|  |  | cg01097881-Rbio | TCACAAAAACCCCACTTTCACTTCCTCTC |
|  |  | cg01097881-S | GGATAGTGTGAGTTTTGAATTGTA |
| WSCD2 | cg13713677 | cg13713677-F | GGAGGGTTAGGTGGTATGT |
|  |  | cg13713677-Rbio | ACCTTACTCCCTCCCCTAAACTTC |
|  |  | cg13713677-S | GGTAGTGTTGTGGTT |
| PDE1C | cg19502018 | cg19502018-Fbio | ATGGTTTATTTAATGGGTATGATAAATTTG |
|  |  | cg19502018-R | ACCCTATCTTTTACCTAAAAAACTCTCA |
|  |  | cg19502018-S | ACCATTATCAACAAACAATT |
| PCDHB15 | cg26380443 | cg26380443-F | AAGGGGAGTGTATAGAAGTAAAGATGG |
|  |  | cg26380443-Rbio | CTCTCTATTTCCTCCATCACA |
|  |  | cg26380443-S | GGTAGGTTGGGAATT |
