# Supplementary material for: Genome-Wide Placental Gene Methylations in Gestational Diabetes Mellitus, Fetal Growth and Metabolic Health Biomarkers in Cord Blood
Source: Front Endocrinol (Lausanne). 2022 May 26;13:875180. doi: 10.3389/fendo.2022.875180 (PMC9204344; doi:10.3389/fendo.2022.875180)
Supplement: Supplementary file 1 [file DataSheet_1.zip › Supplementary Figures_ S1_S2.DOCX]

**Figure S1.** Density plots of beta values in GDM and euglycemic (control) groups in a placental DNA genome-wide methylation association study. GDM=gestational diabetes mellitus.


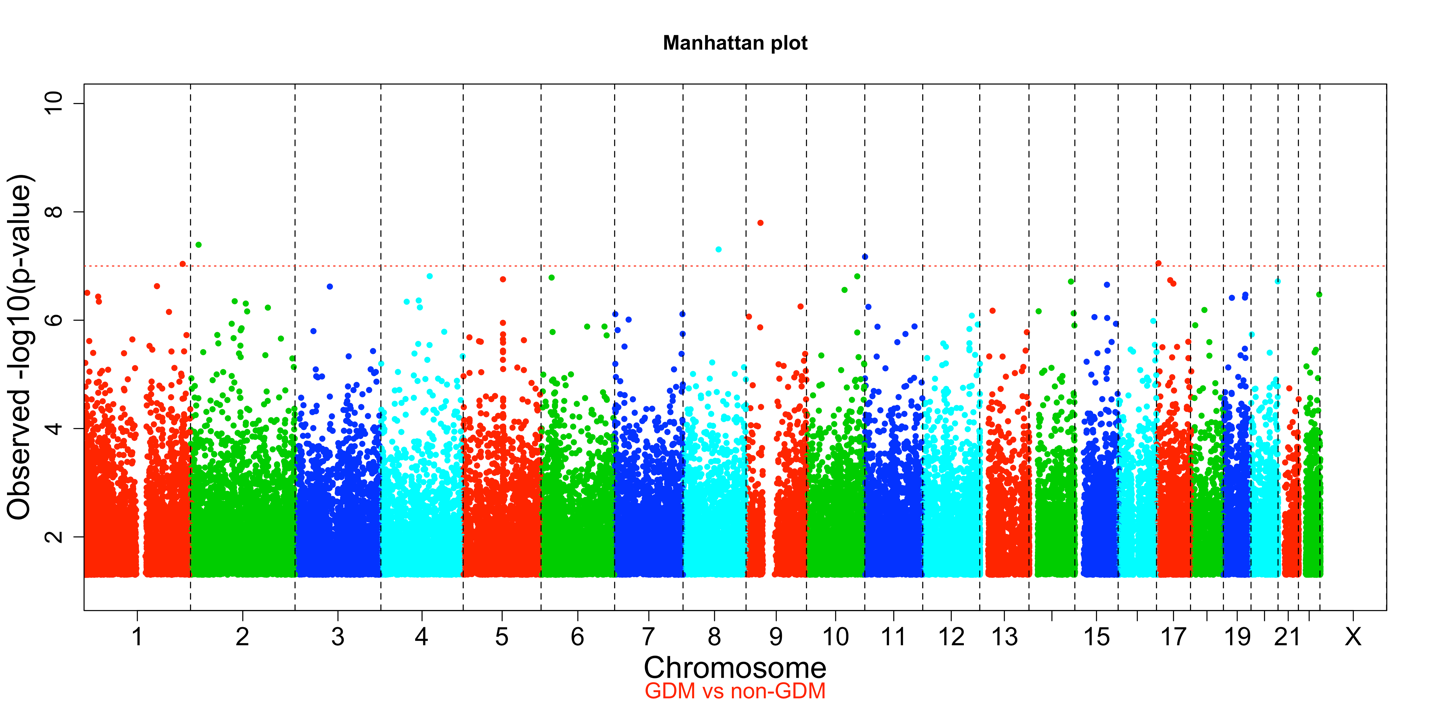


Figure S2. Manhanttan plot in an epigenome wide association study (EWAS) comparing placental DNA methylations in GDM vs. non-GDM (euglycemic) pregnancies
