# Supplementary material for: Genome-Wide Placental Gene Methylations in Gestational Diabetes Mellitus, Fetal Growth and Metabolic Health Biomarkers in Cord Blood
Source: Front Endocrinol (Lausanne). 2022 May 26;13:875180. doi: 10.3389/fendo.2022.875180 (PMC9204344; doi:10.3389/fendo.2022.875180)
Supplement: Supplementary file 1 [file DataSheet_1.zip › Supplementary Tables_S1_S2_S4_S6.DOCX]

**Table S1**. Placental gene DNA CpG site methylation levels (%) in an independent pyrosequencing validation study (n=47 pairs of GDM and controls) vs. the corresponding results in the genome-wide association study (n=30 pairs of GDM and controls) using the Infinium MethylationEPIC Beadchip (850K)

|  |  | **Pyrosequencing** | | |  | **850K** | | |
| --- | --- | --- | --- | --- | --- | --- | --- | --- |
|  |  | GDM (n=47) | Control (n=47) | *P |  | GDM (n=30) | Control (n=30) | *P |
|  |  | Mean±SD | Mean±SD |  |  | Mean±SD | Mean±SD |  |
| cg01097881 | WSCD2 | 20.52±11.4 | 22.77±11.6 | 0.637 |  | 24.52±22.46 | 36.68±14.57 | 8.31e-06 |
| cg13713677 | WSCD2 | 18.8±11.9 | 19.9±14.4 | 0.852 |  | 18.17±17.73 | 33.48±16.73 | 3.63e-05 |
| cg19502018 | PDE1C | 54.0±9.15 | 55.9±12.0 | 0.607 |  | 58.37±7.83 | 69.41±8.59 | 4.47e-07 |
| cg26380443 | PCDHB15 | 17.0±11.4 | 15.6±13.5 | 0.700 |  | 38.60±11.51 | 21.08±13.33 | 4.02e-05 |

*Adjusting for maternal factors (age, pre-pregnancy BMI, parity and delivery mode), infant factors (sex and gestational age at birth) and placental cell type heterogeneity. All the differences between GDM and control groups were in the same direction in the pyrosequencing validation vs. 850K discovery study.

**Table S2**. Validated differentially methylated placental genes in GDM vs controls

| Gene | **This study** | | | **The literature** | | |
| --- | --- | --- | --- | --- | --- | --- |
|  | CpG site | Gene group | Delta beta | CpG site ^ref*^ | Gene group | Delta beta |
| **Hypermethylated** |  |  |  |  |  |  |
| CYP2D7P1 | cg26169700 | Body | 0.057 | cg12850546^13^ | Body | 0.13 |
| GFRA1 | cg06039355 | 5'UTR;TSS1500 | 0.12 | cg12664951^13^ | 3'UTR | 0.11 |
| GFRA1 | cg27034576 | 5'UTR;1stExon | 0.08 |  |  |  |
| HDAC4 | cg14020052 | Body | 0.054 | cg08239041^13^ | Body | 0.13 |
| LIMS2 | cg19326543 | Body;TSS1500;  5'UTR | 0.074 | cg01201482^13^ | Body;5'UTR | 0.30 |
| NAV3 | cg20297402 | Body | 0.064 | cg15405572^16^ | TSS1500 | 0.058 |
| PAX6 | cg09382096 | Body | 0.092 | cg16865446^16^ | Body | 0.098 |
| UPK1B | cg26433505 | Body | 0.055 | cg02019333^13^ | TSS1500 | 0.11 |
| **Hypomethylated** |  |  |  |  |  |  |
| DPP10 | cg16516973 | Body | -0.067 | cg11731746^11^ | Body | -0.088 |
| DPP10 | cg16863522 | 3'UTR | -0.180 |  |  |  |
| DPP10 | cg05581275 | Body | -0.115 |  |  |  |
| CPLX1 | cg01322142 | Body | -0.065 | cg04048250^13^ | Body | -0.11 |
| CSMD2 | cg19081259 | ExonBnd;Body | -0.095 | cg26150071^13^ | Body | -0.10 |
| GPR133 | cg10893220 | Body | -0.071 | cg21046080^13^ | Body | -0.22 |
| NRXN1 | cg13594075 | TSS200 | -0.114 | cg16852792^16^ | Body | -0.069 |
| PCSK9 | cg25957967 | TSS200 | -0.052 | cg00045070^13^ | TSS1500 | -0.21 |
| PCSK9 | cg09072162 | TSS200 | -0.068 |  |  |  |
| PCSK9 | cg17167852 | TSS200 | -0.076 |  |  |  |
| PENK | cg27531336 | TSS1500 | -0.157 | cg11060276^11^ | TSS1500;TSS200 | -0.065 |
| PRDM16 | cg23448811 | Body | -0.092 | cg04493316^11^ | Body | -0.112 |
| PTPRN2 | cg02041981 | Body | -0.060 | cg08490529^11^ | Body | -0.120 |
| PTPRN2 | cg24764310 | Body | -0.056 | cg23162598^13^ | Body | -0.16 |
|  |  |  |  | cg27392792^13^ | TSS1500 | -0.14 |
|  |  |  |  | cg11293572^13^ | Body | -0.13 |
|  |  |  |  | cg21232767^13^ | Body | -0.12 |
|  |  |  |  | cg10641986^16^ | TSS200 | -0.115 |
|  |  |  |  | cg11598005^16^ | Body | -0.056 |
|  |  |  |  | cg05116906^16^ | TSS200 | -0.052 |
| TNXB | cg18460422 | Body | -0.055 | cg10365886^16^ | Body | -0.072 |

*Ref: the cited reference number

**Table S4.** Placental DNA differentially methylated regions (DMR) identified by Comb-p package in R*

| **DMR** | **850k CpGs** | **Direction of association** | **Sidák Corrected Region P-Value** | **Gene** | **Gene group/**  **Relation to Island** |
| --- | --- | --- | --- | --- | --- |
| Chr1:55505148-55505190 | cg25957967,  cg09072162,  cg17167852 | - | 3.85E-05 | PCSK9 | TSS200/Island |
| Chr5:92275335-92275388 | cg21937916,  cg19564029 | + | 2.93E-05 | NA | OpenSea |
| Chr9:33380580-3338581 | cg05640422 | - | 0.008 | NA | OpenSea |
| Chr12:108523395-108523473 | cg01097881,  cg13236378,  cg05062612,  cg26373942,  cg06326926 | - | 5.36E-10 | WSCD2 | TSS200/Island |

NA: not applicable

The comb-p analysis was conducted on the results from robust linear regression models of the association between gestational diabetes mellitus (exposure) and methylation level at each CpG site (outcome), adjusting for maternal age, pre-pregnancy BMI, infant sex, gestational age at birth, primiparity, mod of delivery (cesarean section/vaginal) and estimated cell type components (based on principal component analysis).

**Table S6.** Gene specific correlations between placental DNA methylations and cord blood biomarkers

| Biomarker | CpG sites | Beta | r | P^a^ | P^b^ | UCSC_RefGene_Name | UCSC_RefGene_Group |
| --- | --- | --- | --- | --- | --- | --- | --- |
|  |  | Mean±SD |  |  |  |  |  |
| Leptin | cg05136031 | 0.27±0.04 | 0.28 | 0.033 | 0.56 | LEP | TSS1500 |
| Insulin | cg17434309 | 0.27±0.10 | 0.32 | 0.009 | 0.64 | INS-IGF2;IGF2AS;IGF2 | Body;TSS1500; 5'UTR; |
| Insulin | cg10650127 | 0.65±0.03 | 0.29 | 0.028 | 0.95 | IGF2;INS-IGF2 | TSS200;Body |
| Proinsulin | Mean of cg27331871, cg25742037 | 0.13±0.03 | -0.27 | 0.042 | 0.99 | IGF2;INS-IGF2 | Body;TSS1500 |
| Proinsulin | cg13670288 | 0.58±0.05 | -0.26 | 0.047 | 0.99 | INS-IGF2 | Body |
| C-peptide | cg17434309 | 0.27±0.10 | 0.28 | 0.034 | 0.97 | INS-IGF2;IGF2AS;IGF2 | Body;TSS1500; 5'UTR |
| IGF2 | cg23889607 | 0.60±0.04 | 0.34 | 0.009 | 0.64 | INS-IGF2;IGF2 | Body;3'UTR; |
| IGF2 | Mean of cg02749887,  cg21574853 | 0.35±0.04 | -0.30 | 0.025 | 0.73 | INS-IGF2;INS | TSS200 |
| IGF2 | cg24366657 | 0.10±0.08 | 0.28 | 0.033 | 0.73 | INS-IGF2;IGF2AS;IGF2 | TSS1500; 5'UTR |
| IGF2 | cg25163476 | 0.09±0.06 | 0.27 | 0.041 | 0.73 | INS-IGF2;INS | TSS200 |

^a^P value of partial correlation coefficient adjusting for gestational age at birth.

^b^P value with Benjamini-Hochberg correction for multiple tests.
